# Supplementary material for: DNMT and HDAC inhibition induces immunogenic neoantigens from human endogenous retroviral element-derived transcripts
Source: Nat Commun. 2023 Oct 23;14:6731. doi: 10.1038/s41467-023-42417-w (PMC10593957; doi:10.1038/s41467-023-42417-w)
Supplement: Supplementary file 1 — Supplementary Information [file 41467_2023_42417_MOESM1_ESM.docx]

**DNMT and HDAC inhibition induces immunogenic neoantigens from human endogenous retroviral element-derived transcripts**

Ashish Goyal^1,*^, Jens Bauer^2,3,4,*^, Joschka Hey^1,5,6,*^, Dimitris N Papageorgiou^7,8^, Ekaterina Stepanova^9^, Michael Daskalakis^1,14^, Jonas Scheid^2,3,4,10^, Marissa Dubbelaar^2,3,4,10^, Boris Klimovich^4,11^, Dominic Schwarz^7^, Melanie Märklin^4,11^, Malte Roerden^2,4^, Yu-Yu Lin^1^, Tobias Ma^12^, Oliver Mücke^1^, Hans-Georg Rammensee^3,4,13^, Michael Lübbert^12^, Fabricio Loayza-Puch^9^, Jeroen Krijgsveld^7,8^, Juliane S. Walz^2,3,4,11,#^, Christoph Plass^1,6,13,#^

^1^Cancer Epigenomics, German Cancer Research Center (DKFZ), Heidelberg, Germany

^2^Department of Peptide-based Immunotherapy, University of Tübingen and University Hospital Tübingen, Tübingen, Germany

^3^Institute for Cell Biology, Department of Immunology, University of Tübingen, Tübingen, Germany

^4^Cluster of Excellence iFIT (EXC2180) “Image-Guided and Functionally Instructed Tumor Therapies”, University of Tübingen, Tübingen, Germany

^5^German-Israeli Helmholtz Research School in Cancer Biology, Heidelberg, Germany

^6^German Center for Lung Research, (DZL) partner site Heidelberg, Heidelberg, Germany

^7^Division of Proteomics of Stem Cells and Cancer, German Cancer Research Center (DKFZ), Heidelberg, Germany

^8^Heidelberg University, Medical Faculty, Heidelberg, Germany

^9^Translational Control and Metabolism, German Cancer Research Center (DKFZ), Heidelberg, Germany

^10^Quantitative Biology Center (QBiC), University of Tübingen, Tübingen, Germany

^11^Clinical Collaboration Unit Translational Immunology, German Cancer Consortium (DKTK), Department of Internal Medicine, University Hospital Tübingen, Tübingen, Germany

^12^Department of Hematology, Oncology and Stem Cell Transplantation, University Medical Center Freiburg, Faculty of Medicine, University of Freiburg, Freiburg, Germany

^13^German Cancer Consortium (DKTK), Heidelberg, Germany

^14^Present affiliation: Department of Hematology and Central Hematology Laboratory, Inselspital, Bern, University Hospital, University of Bern, Switzerland

**^*^These authors contributed equally**

**^#^These authors jointly supervised this work**

**Supplementary Figures 1 to 10:**

**Supplementary Fig.1. Comparison of RNA-seq and CAGE-seq data**

**Supplementary Fig.2. Assessment of the de novo transcriptome assembly**

**Supplementary Fig.3. Validation of t-neopeptides via comparative mass spectra**

**Supplementary Fig.4. Single t-neopeptide might originate from multiple transcripts**

**Supplementary Fig.5. P_A*24_ elicits T cell responses**

**Supplementary Fig.6. ERV-derived treatment responses are conserved across cancer entities**

**Supplementary Fig.7. Annotation and validation of t-neopeptides in AML patients via comparative mass spectra**

**Supplementary Fig.8. Differential expression analysis of known transcripts.**

**Supplementary Fig.9.** **t-neopeptides arise from small ORFs which are not detected in whole-cell proteomics analysis.**

**Supplementary Fig.10. Gating strategies applied for the analyses of flow cytometry-acquired data**

**Supplementary Table S1: HLA typing of healthy volunteers.**

**
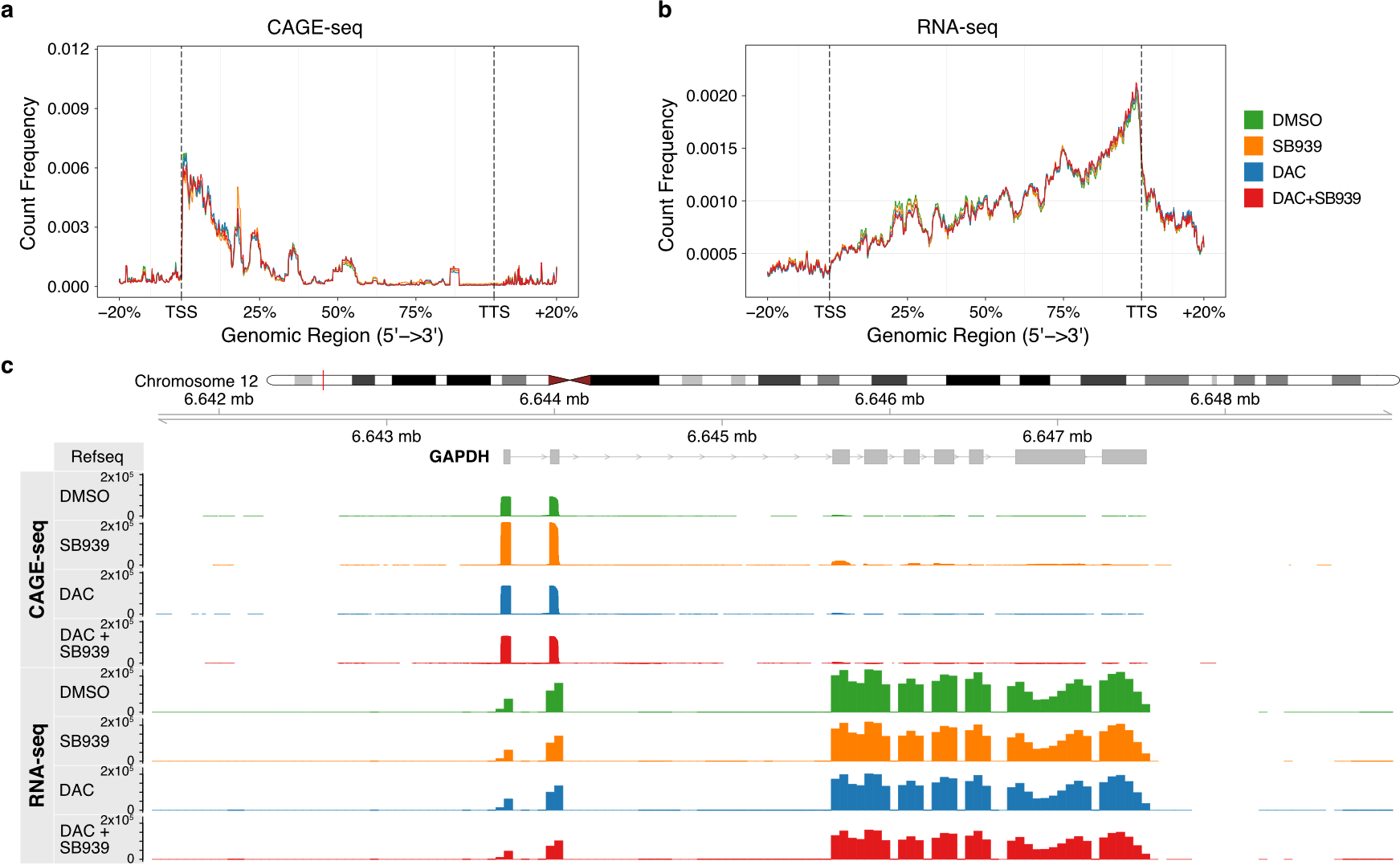
**

**Supplementary Figure 1: Comparison of RNA-seq and CAGE-seq.** (**a** and **b**) Gene body coverage from transcriptional start sites (TSS) to transcriptional termination sites (TTS) across all known genes for (**a**) CAGE-seq and (**b**) RNA-seq data, respectively. (**c**) Locus plot of the GAPDH locus showing (from top to bottom) the GENECODE transcriptome assembly, normalized CAGE-seq, and RNA-seq coverage of DMSO, SB939, DAC, and DAC + SB939 treated NCI-H1299 cells.

**
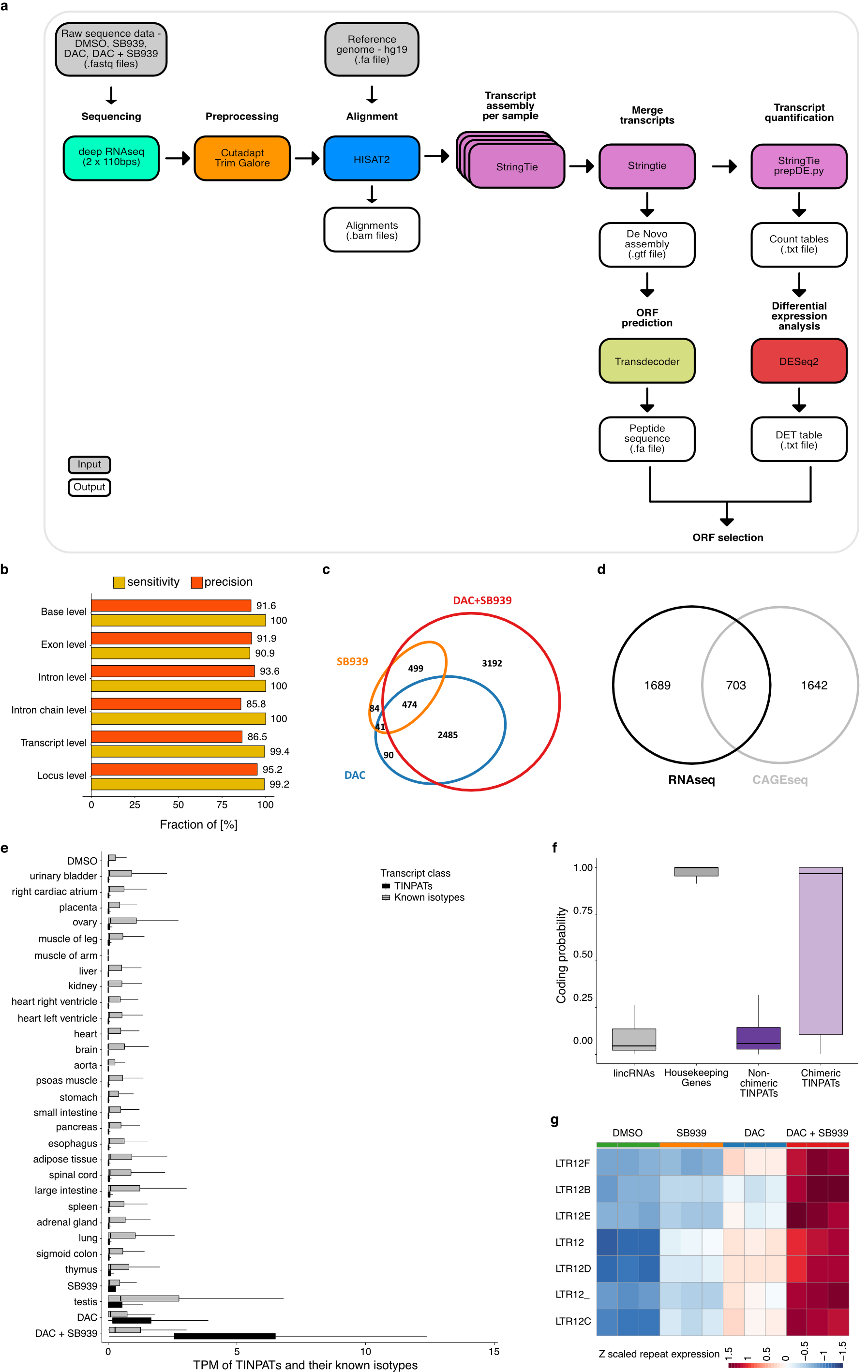
**

**Supplementary Figure 2: Assessment of the de novo** **transcriptome assembly.** (**a**) Graphical representation of the de novo transcriptome assembly workflow using RNA-seq data of DMSO, SB939, DAC, and DAC + SB939 treated NCI-H1299 cells**.** (**b**) Sensitivity and precision of the de novo as compared to the GENCODE transcriptome assembly on base, exon, intron, intron chain, transcript, and locus level. (**c**) Intersection of treatment-induced novel poly-adenylated transcripts (TINPATs; adjusted *p*-value < 0.01, log_2_ fold change >2, classified as chimeric or non-chimeric (novel) transcripts) in the SB939 vs DMSO, DAC vs DMSO, and DAC + SB939 vs DMSO comparison. (**d**) Intersection of transcription start sites of TINPATs (identified using RNAseq) with previously identified treatment-induced non-annotated transcription start sites (identified using CAGEseq). (**e**) Transcripts per million (TPM) of TINPATs, as well as their known isotypes (transcripts that were annotated to the same gene locus as TINPATs) in DMSO, SB939, DAC, and DAC+SB939-treated NCBI-H1299 cells, as well as diverse human tissues. Human tissue data was acquired from ENCODE. (**f**) Coding probability of chimeric and non-chimeric TINPATs as well as lincRNAs and housekeeper genes. Box plots indicate the largest value within the 1.5 times interquartile range above 75^th^ percentile, 75^th^ percentile, median, 25^th^ percentile, and smallest value within the 1.5 times interquartile range below 25^th^ percentile. (**g**) Expression of different LTR12 subfamilies in DMSO, SB939, DAC, and DAC+SB939-treated NCBI-H1299 cells, quantified via RNA-seq. Z-scaled expression was applied to hierarchical clustering.

**
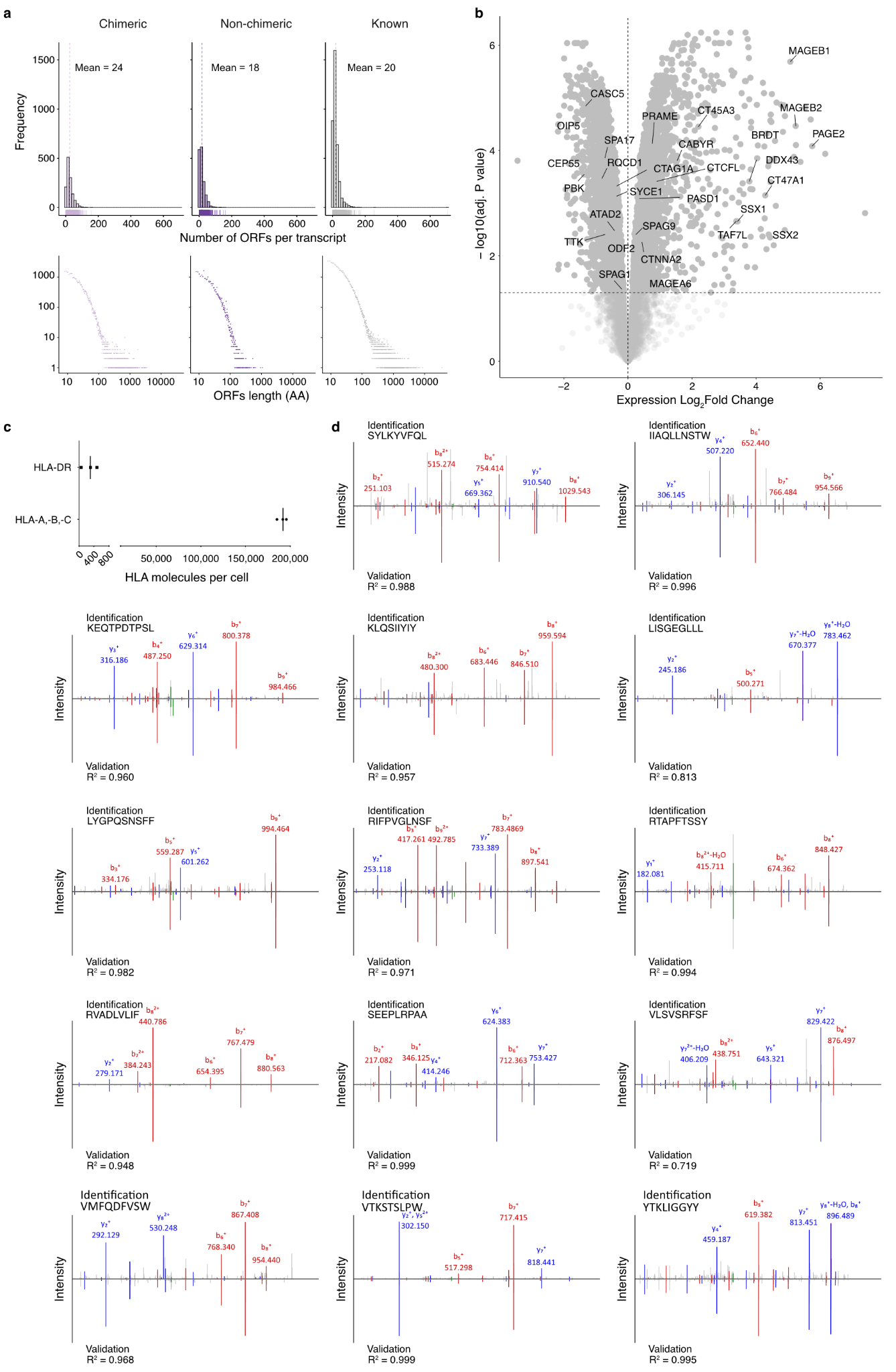
**

**
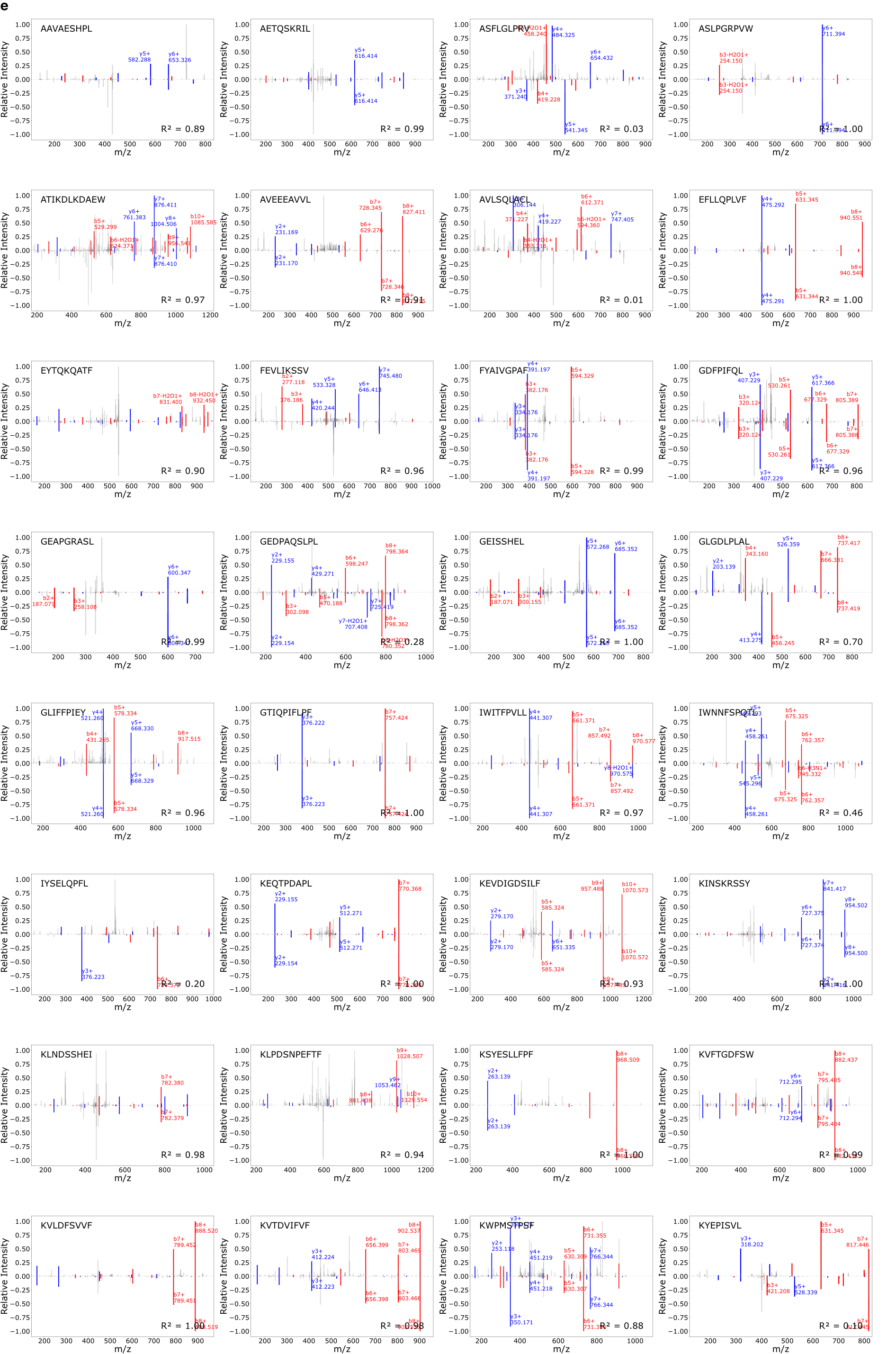
**

**
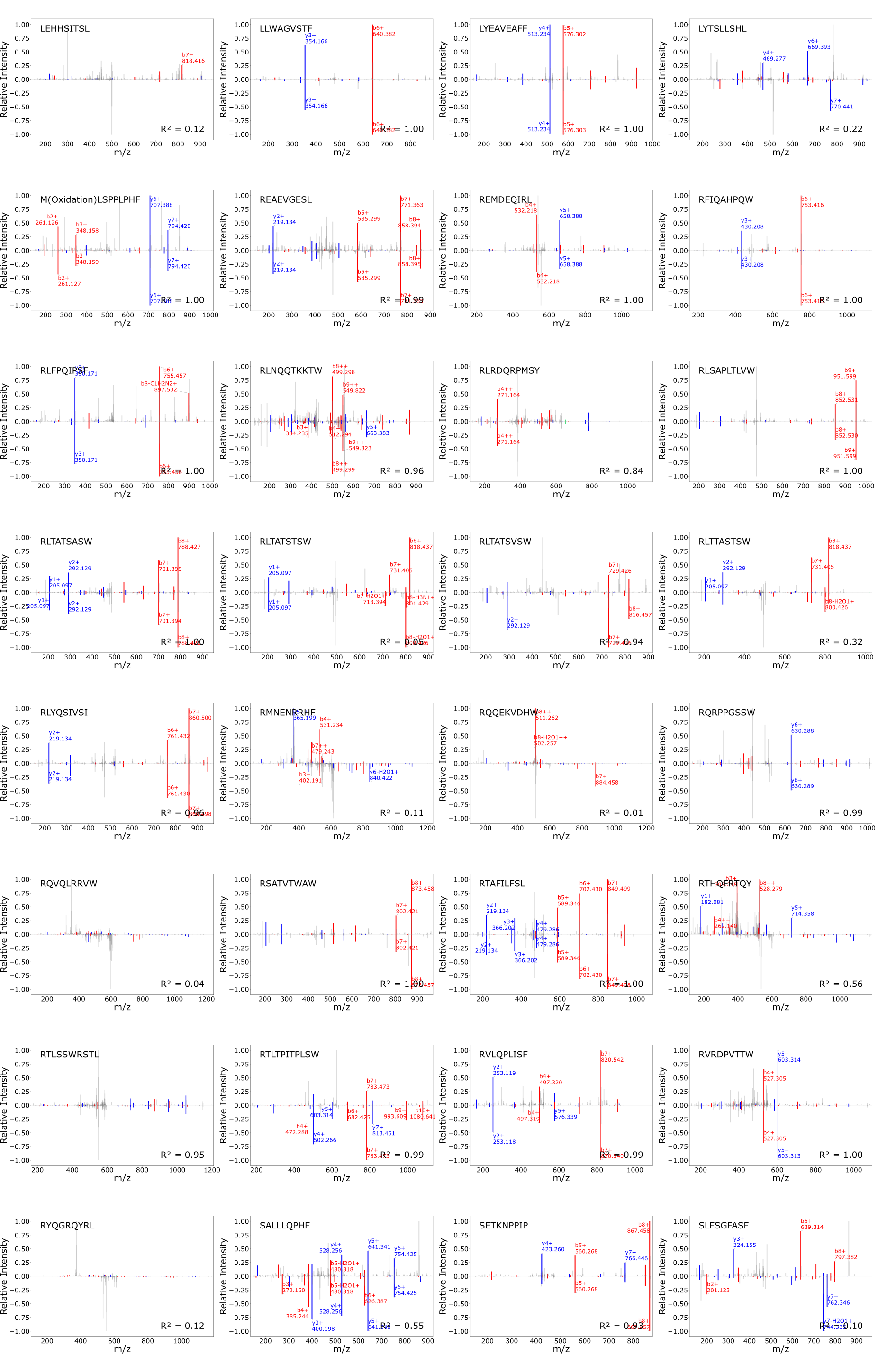
**

**
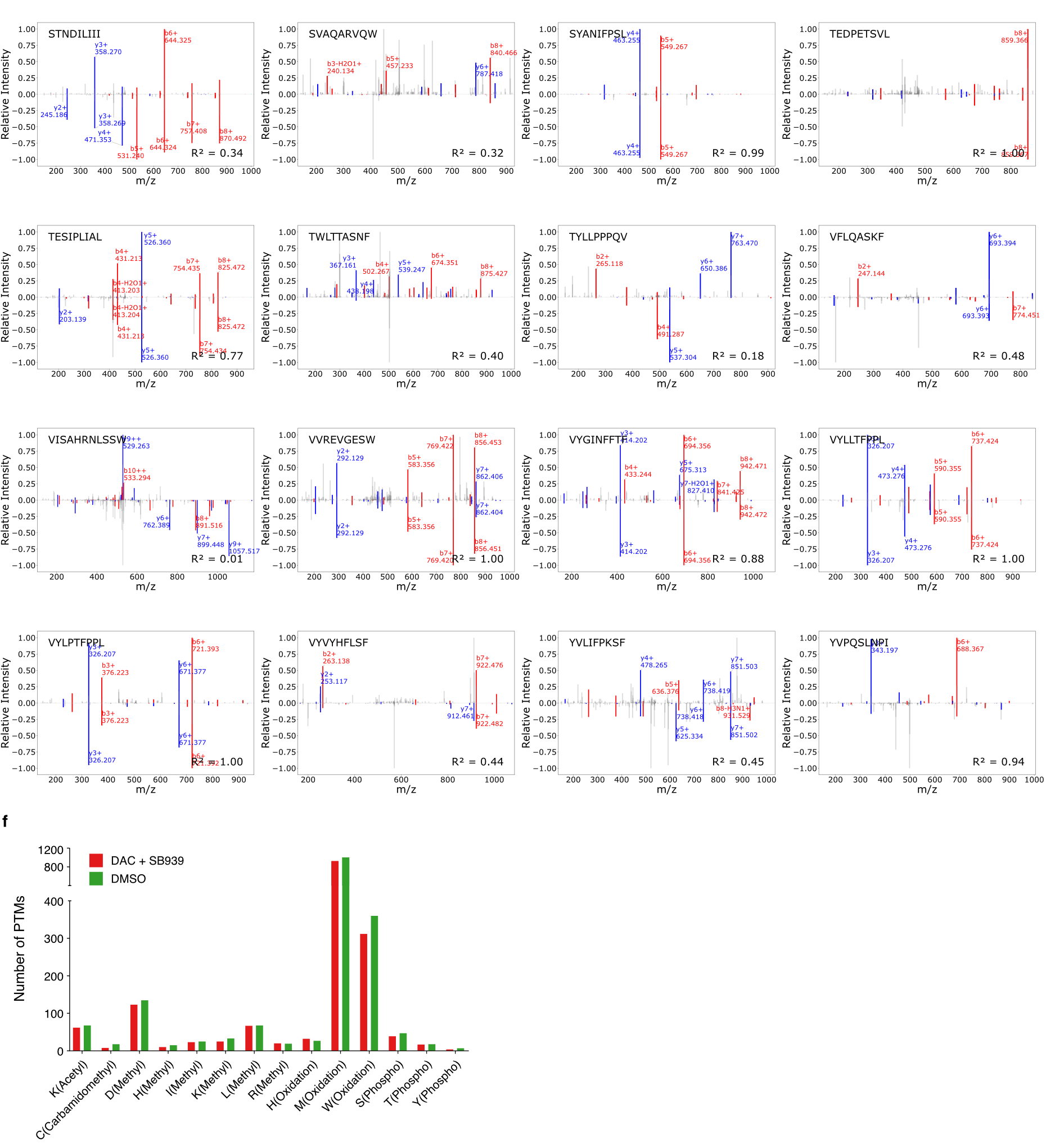
**

**Supplementary Figure 3: Validation of t-neopeptides via comparative mass spectra.** (**a**) Frequency distribution of the number of ORFs per transcript (top) and length distribution of the predicted ORFs (bottom) stratified by transcript classification in known, chimeric, and non-chimeric transcripts. (**b**) Volcano plot of differentially expressed proteins that were detected upon DAC + SB939 treatment using the AHA pulse SILAC labeling approach (**c**) HLA surface expression for HLA-A, -B, -C, and HLA-DR of H1299 cells. (**d**) Fragment spectra comparison (m/z on the x-axis) of the experimentally eluted DAC + SB-induced novel ORF-derived HLA class I-presented ligands SYLKYVFQL (P_A*24_), IIAQLLNSTW, KEQTPDTPSL, KLQSIIYIY, LISGEGLLL, LYGPQSNSFF, RIFPVGLNSF, RTAPFTSSY, RVADLVLIF, SEEPLRPAA, VLSVSRFSF, VMFQDFVSW, VTKSTSLPW, and YTKLIGGYY identified in all three replicates of DAC + SB939 treated H1299 cells (identification) to the respective synthetic peptide (validation, mirrored on the x-axis) with the calculated spectral correlation coefficient (R^2^). Identified b- and y-ions are marked in red and blue, respectively (**e**) Fragment spectra comparison (m/z on the x-axis) of all remaining experimentally eluted ORF-derived HLA class I-presented ligands extracted from H1299 cells (identification) to the respective synthetic peptide, if the sequence match was possible (n=80, validation, mirrored on the x-axis) with the calculated spectral correlation coefficient (R^2^). Identified b- and y-ions are marked in red and blue, respectively. (**f**) Comparison of the amount of individual PTMs (oxidation of methionine, tryptophan, and histidine, methylation of aspartic acid, histidine, isoleucine, leucine, lysine, and arginine, acetylation of lysine, carbamidomethylation of cysteine, and phosphorylation of serine, threonine, and tyrosine) identified in the PTM analysis of the immunopeptidomes of DAC + SB939 treated (n=3) and DMSO control cells (n=3).

**
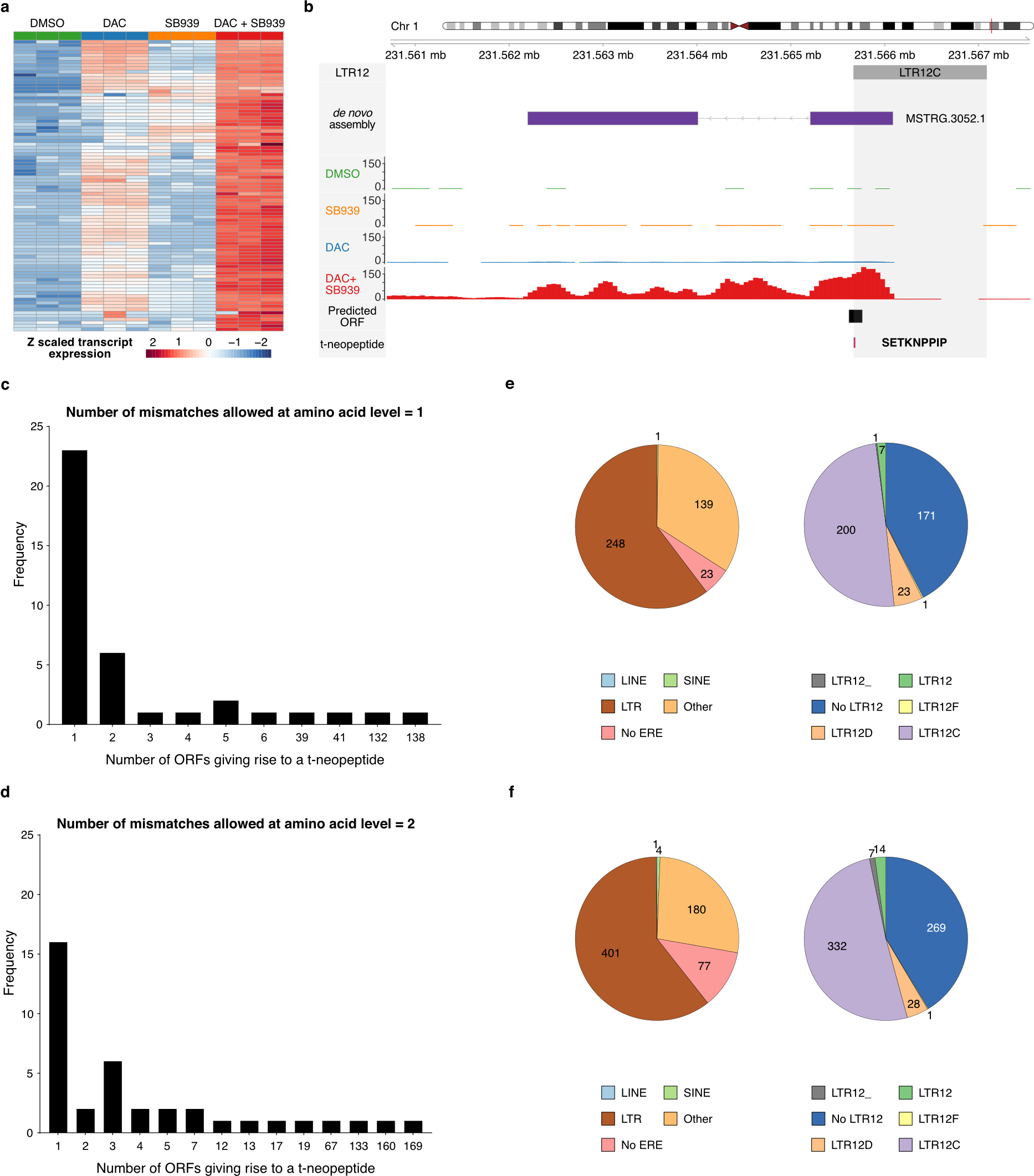
**

**Supplementary Figure 4: Single t-neopeptide might originate from multiple transcripts.** (**a**) Expression of 88 transcripts that were assigned to t-neopeptide encoded within LTR12C repeat. Z-scaled expression was applied to hierarchical clustering. (**b**) Locus plot of a selected treatment-induced novel polyA+ transcript (TINPAT) giving rise to t-neoantigen within LTR12C repeat, showing (from top to bottom) LTR12 repeats, the de novo transcriptome assembly, normalized RNA-seq coverage of DMSO, SB939, DAC, and DAC + SB939 treated NCI-H1299 cells, predicted ORF, and corresponding t-neoantigen SETKNPPIP. (**c**) and (**d**) Frequency distribution of the number of matches with UniProt human protein database or DAC + SB939 induced ORFs, allowing for (**c**) 1, or (**d**) 2 mismatches with t-neopeptides. (**e**) and (**f**) The number of transcriptional start sites of transcripts that give rise to t-neopeptides allowing for (**e**) 1, or (**f**) 2 mismatches and overlap with transposable element families.

**
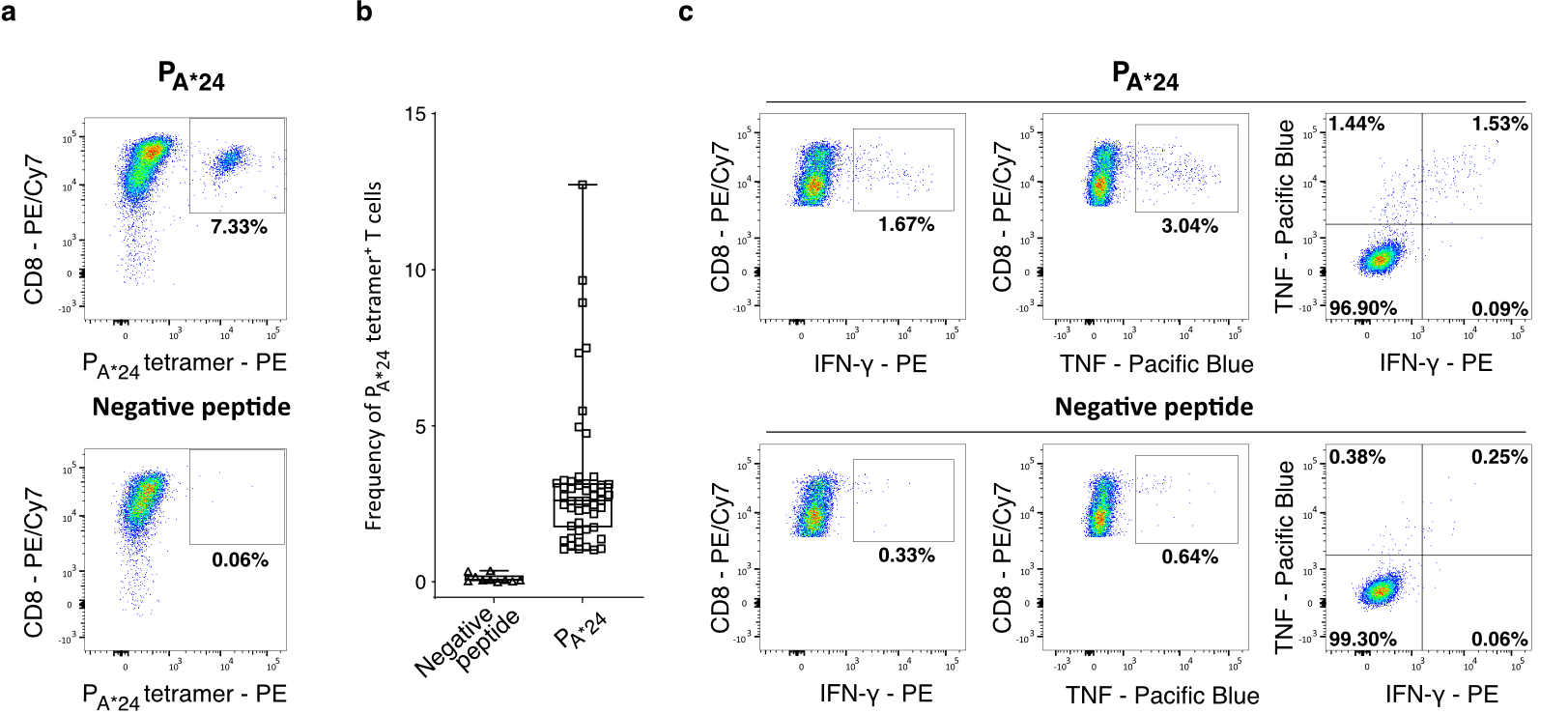
**

**Supplementary Figure 5: P_A*24_ elicits T cell responses.** (**a**) Representative example of flow cytometry-based characterization of P_A*24_-specific CD8^+^ T cells of a healthy volunteer (HV) after *in vitro* artificial antigen-presenting cell (aAPC)-priming with HLA-A*24-P_A*24_-monomer. (**b**) Frequencies of P_A*24_-specific CD8^+^ T cells compared to CD8^+^ T cells primed with an HLA-matched negative peptide in HVs (n = 3, frequency of T cells is indicated per well). All data points are shown, the band indicates the median, and the box indicates the first and third quartiles. (**c**), Representative example of IFN-γ and TNF production of P_A*24_-specific CD8^+^ T cells stimulated with P_A*24_ (upper panel) or an HLA-matched negative peptide (lower panel) after aAPC-priming.


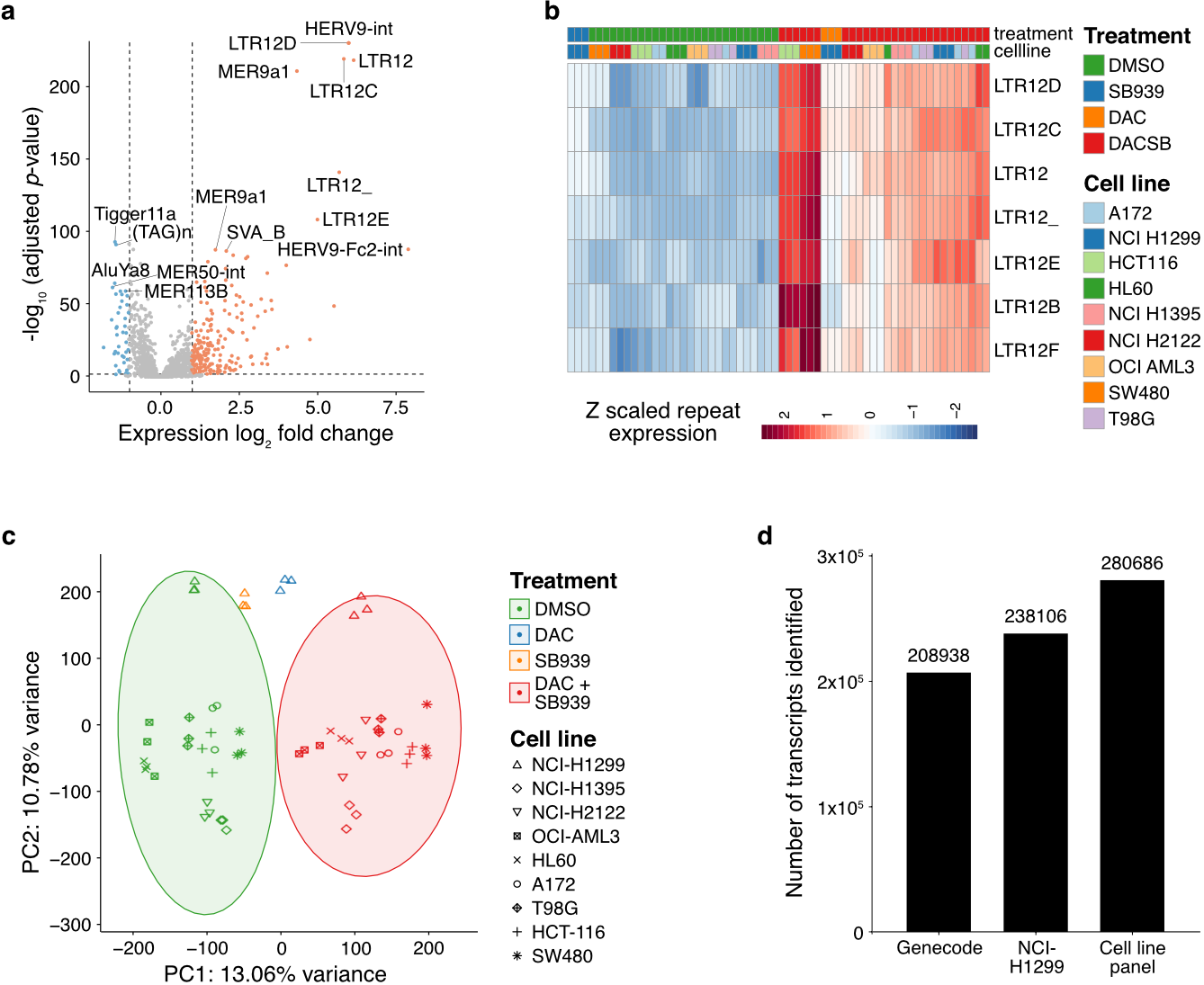


**Supplementary Figure 6: ERV-derived treatment responses are conserved across cancer entities.** (**a**) Volcano plot of differential transposable element expression analyses, comparing DAC + SB939 vs DMSO. Differentially expressed transposable elements are defined by DESeq2: adjusted *p*-value < 0.05, absolute log_2_ fold change >1. (**b**) Expression of different LTR12 subfamilies in the DMSO and DAC + SB939 treated cell line panel, as well as DMSO, SB939, DAC, and DAC+SB939-treated NCBI-H1299 cells. Z-scaled expression was applied to hierarchical clustering. (**c**) Principal component (PC) analysis of the 5,000 most variably expressed transcripts of DAC + SB939 (n = 3) and DMSO-treated (n = 3) cancer cell lines, assessed via RNA-seq. Transcript expression was quantified using the de novo transcriptome assembly of all cell lines. PC1 and PC2 are visualized. (**d**) The number of transcripts identified in the GENECODE, NCI-H1299, and cell line panel de novo transcriptome assembly.


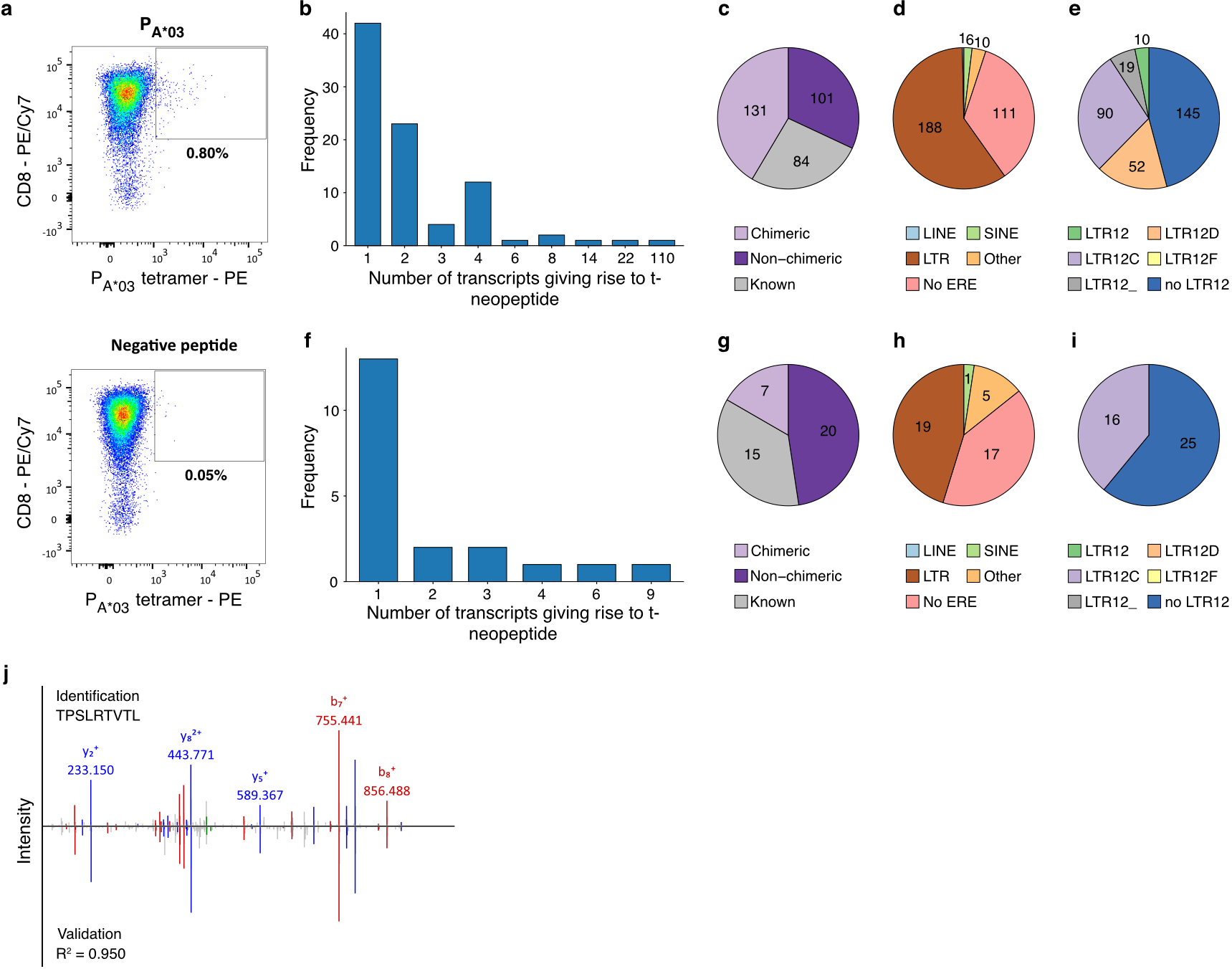


**Supplementary Figure 7: Annotation and validation of t-neopeptides in AML patients via comparative mass spectra.** (**a**) Flow cytometry-based characterization of RTDSSLLEK (P_A*03_)-specific CD8+ T cells of a healthy volunteer (HV) after in vitro artificial antigen-presenting cell (aAPC)-priming with HLA-A*03-P_A*03_-monomer. (**b** and **f**) The number of DAC-induced transcripts giving rise to t-neopeptides, identified in AML patients’ (**b**) HLA class I and (**f**) HLA class II immunopeptidomics, respectively. (**c** and **g**) Classification of transcripts giving rise to t-neopeptides, presented by (**c**) HLA class I and (**g**) HLF class II, in known, chimeric, and non-chimeric novel transcripts. (**d, e, h,** and **i**) The number of transcriptional start sites of transcripts that give rise to t-neopeptides, presented by (**d** and **e**) HLA class I and (**h** and **i**) HLA class II, and overlap with (**d** and **h**) transposable element families and (**e** and **i**) LTR12 family**.** (**j**) Fragment spectra comparison (m/z on the x-axis) of the experimentally eluted DAC-induced novel ORF-derived HLA class I-presented ligand TPSLRTVTL extracted from the DAC-treated AML patient uniform patient number (UPN) 2 (identification) to the respective synthetic peptide (validation, mirrored on the x-axis) with the calculated spectral correlation coefficient (R^2^). Identified b- and y-ions are marked in red and blue, respectively.


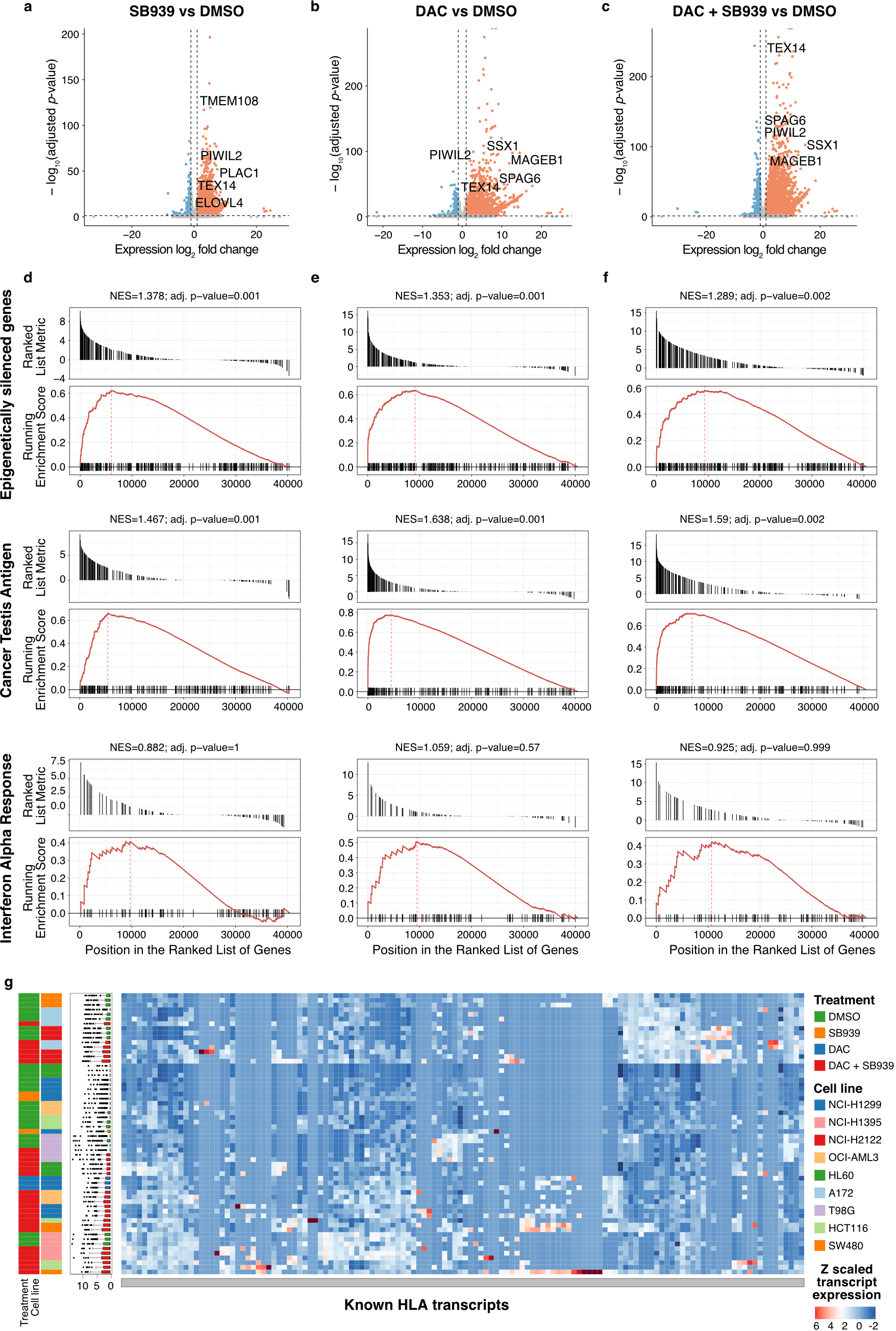


**Supplementary Figure 8: Differential expression analysis of known transcripts**. (**a**, **b**, and **c**) Volcano plot of differential gene expression analyses, comparing (**a**) SB939 vs DMSO, (**b**) DAC vs DMSO, and (**c**) DAC + SB939 vs DMSO. Gene quantification was performed on the known GENECODE transcriptome assembly. Differentially expressed genes are defined by DESeq2: adjusted *P* value < 0.01, absolute log_2_ fold change >2. (**d**, **e,** and **f**) Gene set enrichment analyses of epigenetically silenced (top), cancer-testis antigens (middle), and Interferon-alpha gene (bottom) sets in the (**d**) SB939 vs DMSO, (**e**) DAC vs DMSO, and (**f**) DAC + SB939 vs DMSO comparisons. NES, normalized enrichment score. (**g**) Heat map of z-scaled transcript expression of all known HLA transcripts in the RNAseq dataset of all treated cell lines.

**
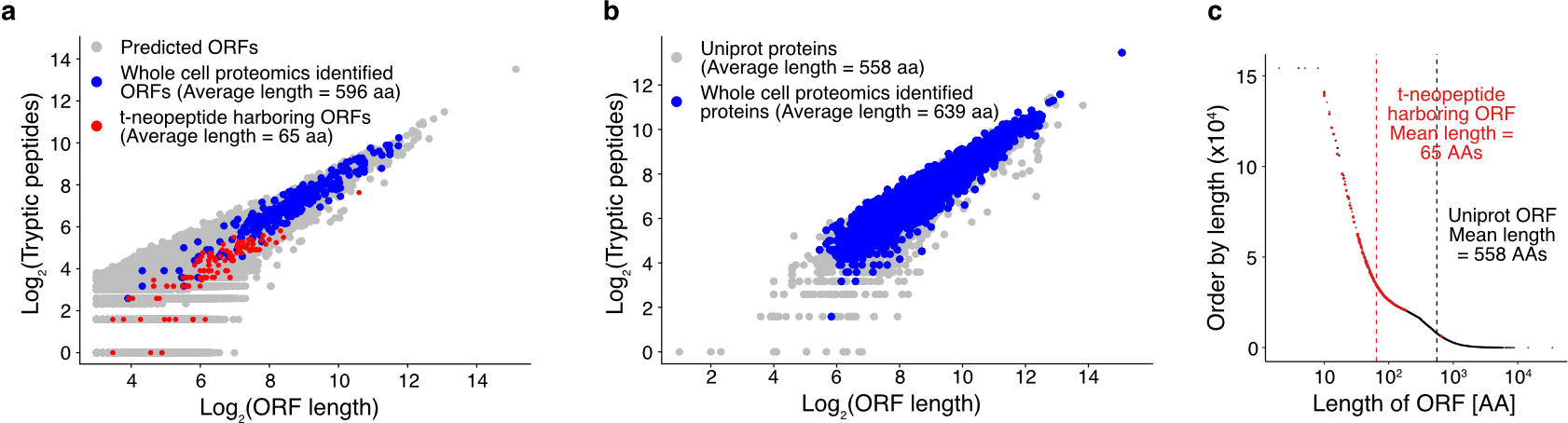
**

**Supplementary Figure 9: t-neopeptides arise from small ORFs which are not detected in whole-cell proteomics analysis**. (**a**) Scatter plot of the number of predicted tryptic peptides versus the open reading frame (ORF) length for all predicted ORFs. All the ORFs identified in whole cell mass spectrometric analysis are highlighted in blue, and all the ORFs predicted to harbor a t-neopeptide are highlighted in red. (**b**) Scatter plot of the number of predicted tryptic peptides versus the open reading frame (ORF) length for all proteins in the Uniprot database. All ORFs identified in whole cell mass spectrometric analysis are highlighted in blue (**c**) Amino acid (AA) length distribution of t-neoepitope harboring ORFs (highlighted in red) as well as UniProt ORFs (highlighted in black).


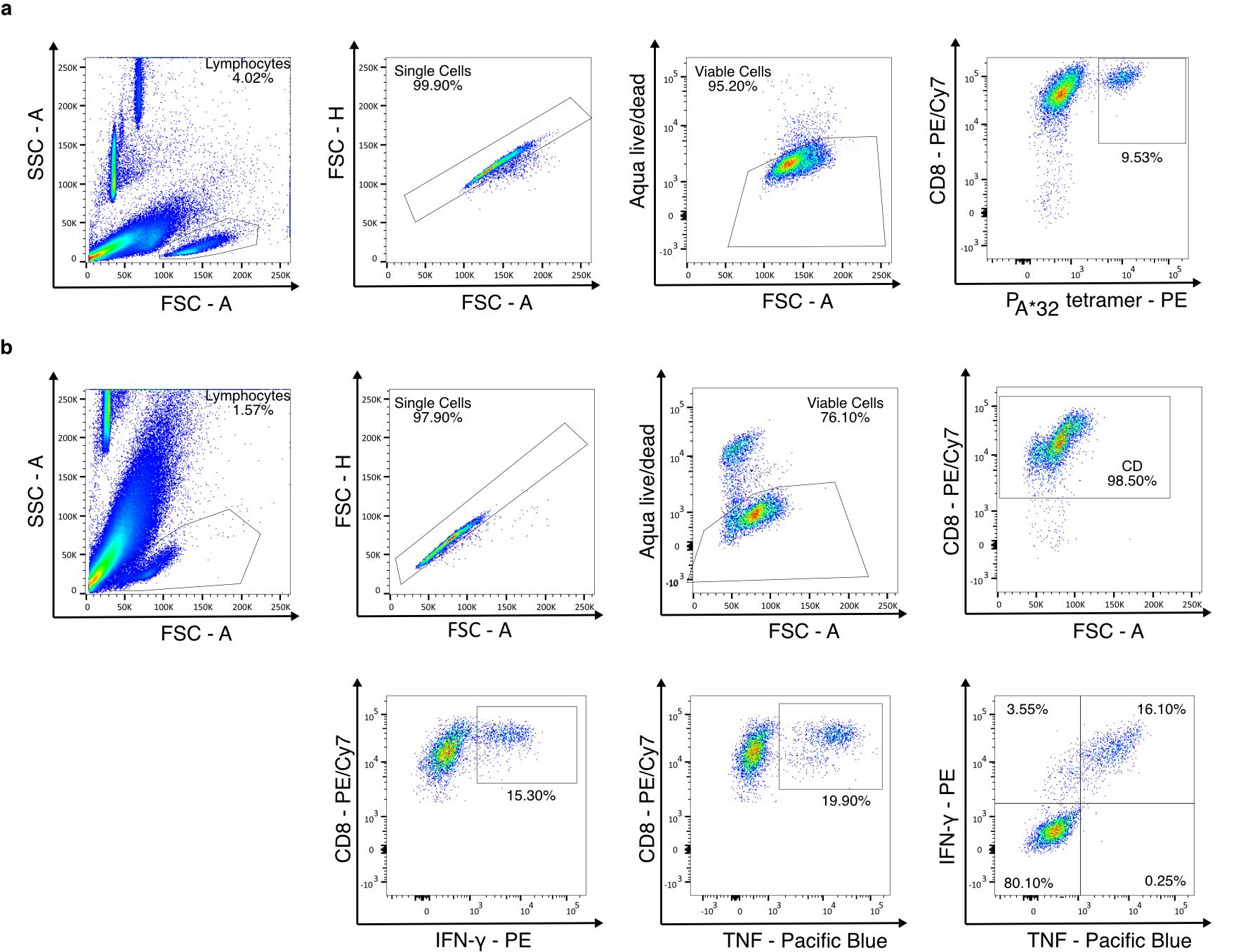


**Supplementary Figure 10: Gating strategies applied for the analyses of flow cytometry-acquired data** (**a**) Exemplary sample showing the gating strategy for intracellular cytokine staining (ICS) of artificial antigen-presenting cell (aAPC)-based CD8+ T cell priming. The first gate identifies the lymphocytes (FSC-A vs. SSC-A), which are further gated for single cells (FSC-A vs. FSC-H), viable cells (FSC-A vs. Aqua live/dead), and CD8+ cells (FSC-A vs. CD8-PE/Cy7). CD8+ T cells were analyzed for IFN-γ (IFN-γ-PE vs. CD8-PE/Cy7), TNF (TNF-Pacific Blue vs. CD8-PE/Cy7), and TNF/ IFN-γ (TNF-Pacific Blue vs. IFN-γ-PE) expression. (**b**) Exemplary sample showing the gating strategy for evaluation of PA*32-tetramer staining of CD8+ T cells after antigen-presenting cell (aAPC)-based priming. The first gate identifies the lymphocytes (FSC-A vs. SSC-A), which are further gated for single cells (FSC-A vs. FSC-H), viable cells (FSC-A vs. Aqua live/dead), and analyzed for CD8+/PA*32-tetramer-specific cells (PA*32 tetramer-PE vs. CD8-PE/Cy7).

**Supplementary Table S1: HLA typing of healthy volunteers.**

| **Donor** | **HLA-A** | **HLA-B** |
| --- | --- | --- |
| HV1 | A*02, A*32 | B*14, B*37 |
| HV2 | A*01, A*32 | B*51, B*44 |
| HV3 | A*23, A*32 | B*38, B*44 |
| HV4 | A*02, A*24 | B*35, B*57 |
| HV5 | A*02, A*24 | B*15, B*44 |

| HV6 | A*03, A*24 | B*15, B*49 |
| --- | --- | --- |
| HV7 | A*03, A*24 | B*07, B*15 |
| HV8 | A*03, A*68 | B*07, B*39 |
| HV9 | A*03, A*31 | B*18, B*35 |

| HV10 | A*02, A*03 | B*07, B*51 |
| --- | --- | --- |

HV, healthy volunteers.
