# Supplementary material for: DNMT and HDAC inhibition induces immunogenic neoantigens from human endogenous retroviral element-derived transcripts
Source: Nat Commun. 2023 Oct 23;14:6731. doi: 10.1038/s41467-023-42417-w (PMC10593957; doi:10.1038/s41467-023-42417-w)
Supplement: Supplementary file 3 — Description of Additional Supplementary Files [file 41467_2023_42417_MOESM3_ESM.pdf]

Description of Additional Supplementary Files

File Name: Supplementary Data 1
Description: De novo transcriptome assembly of NCI-H1299 cells

File Name: Supplementary Data 2
Description: Differential transcript expression results of the de novo assembly

File Name: Supplementary Data 3
Description: TPM of de novo assembled transcripts across different tissues

File Name: Supplementary Data 4
Description: All ORFs predicted from de novo assembly

File Name: Supplementary Data 5
Description: Whole-cell proteomics differential protein abundance

File Name: Supplementary Data 6
Description: Peptides identified via immunopeptidomics of NCI-H1299 cells

File Name: Supplementary Data 7
Description: Novel Peptides identified via immunopeptidomics of NCI-H1299 cells

File Name: Supplementary Data 8
Description: Source ORF mapping of the t-neopeptides identified in NCI-H1299 cells

File Name: Supplementary Data 9
Description: Sequence polymorphism analysis for the t-neopeptides identified in NCI-H1299 cells

File Name: Supplementary Data 10
Description: De novo transcriptome assembly of the cell line panel

File Name: Supplementary Data 11
Description: Differential transcript expression results of the de novo assembly of the cell line panel

File Name: Supplementary Data 12
Description: AML Patient characteristics

File Name: Supplementary Data 13
Description: Peptides identified via immunopeptidomics of AML patients

File Name: Supplementary Data 14
Description: Differential gene expression results of the known assembly (GENECODE)
