# Supplementary material for: DNMT and HDAC inhibition induces immunogenic neoantigens from human endogenous retroviral element-derived transcripts
Source: Nat Commun. 2023 Oct 23;14:6731. doi: 10.1038/s41467-023-42417-w (PMC10593957; doi:10.1038/s41467-023-42417-w)
Supplement: Supplementary file 18 — Reporting Summary [file 41467_2023_42417_MOESM18_ESM.pdf]

nature portfolio

Corresponding author(s): Prof. Dr. Christoph Plass, Prof. Dr. Juliane Walz

Last updated by author(s): 2023/08/30

# Reporting Summary

Nature Portfolio wishes to improve the reproducibility of the work that we publish. This form provides structure for consistency and transparency in reporting. For further information on Nature Portfolio policies, see our Editorial Policies and the Editorial Policy Checklist.

## Statistics

For all statistical analyses, confirm that the following items are present in the figure legend, table legend, main text, or Methods section.

| n/a | Confirmed | |
|---|---|---|
| ☐ | ☒ | The exact sample size (*n*) for each experimental group/condition, given as a discrete number and unit of measurement |
| ☐ | ☒ | A statement on whether measurements were taken from distinct samples or whether the same sample was measured repeatedly |
| ☐ | ☒ | The statistical test(s) used AND whether they are one- or two-sided<br>*Only common tests should be described solely by name; describe more complex techniques in the Methods section.* |
| ☐ | ☒ | A description of all covariates tested |
| ☐ | ☒ | A description of any assumptions or corrections, such as tests of normality and adjustment for multiple comparisons |
| ☐ | ☒ | A full description of the statistical parameters including central tendency (e.g. means) or other basic estimates (e.g. regression coefficient) AND variation (e.g. standard deviation) or associated estimates of uncertainty (e.g. confidence intervals) |
| ☐ | ☒ | For null hypothesis testing, the test statistic (e.g. *F*, *t*, *r*) with confidence intervals, effect sizes, degrees of freedom and *P* value noted<br>*Give P values as exact values whenever suitable.* |
| ☒ | ☐ | For Bayesian analysis, information on the choice of priors and Markov chain Monte Carlo settings |
| ☒ | ☐ | For hierarchical and complex designs, identification of the appropriate level for tests and full reporting of outcomes |
| ☐ | ☒ | Estimates of effect sizes (e.g. Cohen's *d*, Pearson's *r*), indicating how they were calculated |

*Our web collection on statistics for biologists contains articles on many of the points above.*

## Software and code

Policy information about availability of computer code

**Data collection**

Flow cytometric analysis was performed on a FACSCanto II Analyzer (BD Biosciences, New Jersey, USA).

Cytotoxicity analyses were performed using the IncuCyte S3 Live-Cell Analysis System (Sartorius, Göttingen, Germany).

RNAseq libraries were prepared with the TruSeq Stranded mRNA library prep kit from Illumina. Sequencing was performed on a Nextseq 2000, paired-end, 200 bp platform (Illumina).

Whole cell proteomics samples for LC-MS/MS analysis were prepared as described in the methods section, and loaded onto Q Exactive HF Orbitrap LC-MS/MS System (Thermo Fisher Scientific).

For Immunopeptidomics the reversed-phase liquid chromatography nanoUHPLC UltiMate 3000 RSLCnano (ThermoFisher Scientific) and the Orbitrap Fusion Lumos mass spectrometer (ThermoFisher Scientific) were used.

Ribo-seq libraries were prepared as previously described (see Methods section) and sequenced single-end, 100 bp, on one lane of a Nextseq 2000 sequencer (Illumina).

qPCR data were aquired using Lightcycler 480 System (Roche).

**Data analysis**

Flow cytometry data were analysed using FACSDiva software, Cytek Spectroflow and FlowJo 10.4 (BD Biosciences).

Surface marker expression was analysed with the R packages FlowSOM v1.18.0 and ConsensusClusterPlus v1.5

Incucyte data was analysed using the IncuCyte 2020C software.

RNAseq data were processed with the nf-core RNA-seq pipeline v1.2 and the aligner HISAT2 v2.10.

Coverage tracks were generated from bam files utilizing deepTools' v3.3.1 function bamCoverage.

De novo transcriptome assembly: Transcriptomes were assembled using StringTie v2.1.1.

TSS of transcripts were annotated to the closest or overlapping TEs using the R package GenomicRanges v1.38.0.

ORFs were predicted using the software TransDecoder (www.github.com/TransDecoder/TransDecoder) v2.0.

Differential gene expression analysis were performed with the R package DESeq2 v1.26.0

Gene set enrichment analyses were performed utilizing the R package clusterProfiler v3.12.0

Overlap of aligned reads with transposable elements was performed using subread's v1.6.4 function featureCounts.

The R package LOLA v 1.16.0 was used to enrich TSSs with TE classes, families, and subfamilies.

Locus plots were generated using the R package Gviz v1.30.3.
To define the genomic origin of the identified peptide candidates, the originating ORFs were scanned for the identified peptide sequences using Biostring's v3.42.

Custom code for the analysis of next-generation sequencing data was deposited at https://github.com/HeyLifeHD/TINAT.

Whole cell proteomics data was analysed using Maxquant version 2.0.3.0.

Immunopeptidomics data were analyzed using The Proteome Discoverer 1.4.

For manuscripts utilizing custom algorithms or software that are central to the research but not yet described in published literature, software must be made available to editors and reviewers. We strongly encourage code deposition in a community repository (e.g. GitHub). See the Nature Portfolio guidelines for submitting code & software for further information.

# Data

Policy information about availability of data

All manuscripts must include a data availability statement. This statement should provide the following information, where applicable:

- Accession codes, unique identifiers, or web links for publicly available datasets
- A description of any restrictions on data availability
- For clinical datasets or third party data, please ensure that the statement adheres to our policy

The Ribo-seq and RNA sequencing data generated in this study have been deposited in the NCBI Gene Expression Omnibus (GEO) database under the primary accession number GSE209777 (https://www.ncbi.nlm.nih.gov/geo/query/acc.cgi?acc=GSE209777). The whole cell proteomics mass spectrometry data generated in this study have been deposited in the ProteomeXchange Consortium (http://proteomecentral.proteomexchange.org) via the PRIDE 54 partner repository under the dataset identifier PXD035748 (https://proteomecentral.proteomexchange.org/cgi/GetDataset?ID=PXD035748). The immunopeptidomics mass spectrometry data generated in this study have been deposited to the ProteomeXchange Consortium via the PRIDE partner repository under the dataset identifier PXD035750 (https://proteomecentral.proteomexchange.org/cgi/GetDataset?ID=PXD035750).
The H1299 CAGE sequencing publicly available data used in this study are available in the GEO database under accession codes GSE81322 (https://www.ncbi.nlm.nih.gov/geo/query/acc.cgi?acc=GSE81322).
The human tissue RNA sequencing publicly available data used in this study are available in the ENCODE database under accession codes : ENCSR725TPW (https://www.encodeproject.org/experiments/ENCSR725TPW/), ENCSR001UXR (https://www.encodeproject.org/experiments/ENCSR001UXR/), ENCSR612HYR (https://www.encodeproject.org/experiments/ENCSR612HYR/), ENCSR843HXR (https://www.encodeproject.org/experiments/ENCSR843HXR/), ENCSR775KCE (https://www.encodeproject.org/experiments/ENCSR775KCE/), ENCSR629VMZ (https://www.encodeproject.org/experiments/ENCSR629VMZ/), ENCSR635GTY (https://www.encodeproject.org/experiments/ENCSR635GTY/), ENCSR046XHI (https://www.encodeproject.org/experiments/ENCSR046XHI/), ENCSR433XCV (https://www.encodeproject.org/experiments/ENCSR433XCV/), ENCSR071ZMO (https://www.encodeproject.org/experiments/ENCSR071ZMO/), ENCSR693GGB (https://www.encodeproject.org/experiments/ENCSR693GGB/), ENCSR922VBO (https://www.encodeproject.org/experiments/ENCSR922VBO/), ENCSR825GWD (https://www.encodeproject.org/experiments/ENCSR825GWD/), ENCSR686JJB (https://www.encodeproject.org/experiments/ENCSR686JJB/), ENCSR066FZL (https://www.encodeproject.org/experiments/ENCSR066FZL/), ENCSR102TQN (https://www.encodeproject.org/experiments/ENCSR102TQN/), ENCSR547TNE (https://www.encodeproject.org/experiments/ENCSR547TNE/), ENCSR332MTG (https://www.encodeproject.org/experiments/ENCSR332MTG/), ENCSR274JRR (https://www.encodeproject.org/experiments/ENCSR274JRR/), ENCSR721HDG (https://www.encodeproject.org/experiments/ENCSR721HDG/), ENCSR995BHD (https://www.encodeproject.org/experiments/ENCSR995BHD/), ENCSR502OTI (https://www.encodeproject.org/experiments/ENCSR502OTI/), ENCSR917YHC (https://www.encodeproject.org/experiments/ENCSR917YHC/), ENCSR542OHE (https://www.encodeproject.org/experiments/ENCSR542OHE/), ENCSR598KJX (https://www.encodeproject.org/experiments/ENCSR598KJX/), ENCSR880XLM (https://www.encodeproject.org/experiments/ENCSR880XLM/), ENCSR555BCP (https://www.encodeproject.org/experiments/ENCSR555BCP/), ENCSR146ZKR (https://www.encodeproject.org/experiments/ENCSR146ZKR/), ENCSR699YJR (https://www.encodeproject.org/experiments/ENCSR699YJR/), ENCSR675YAS (https://www.encodeproject.org/experiments/ENCSR675YAS/), ENCSR278UYN (https://www.encodeproject.org/experiments/ENCSR278UYN/), ENCSR510PSL (https://www.encodeproject.org/experiments/ENCSR510PSL/), ENCSR763NOO (https://www.encodeproject.org/experiments/ENCSR763NOO/), ENCSR769LNJ (https://www.encodeproject.org/experiments/ENCSR769LNJ/), ENCSR663IOE (https://www.encodeproject.org/experiments/ENCSR663IOE/), ENCSR618IQY (https://www.encodeproject.org/experiments/ENCSR618IQY/), ENCSR993QGR (https://www.encodeproject.org/experiments/ENCSR993QGR/), ENCSR270OKS (https://www.encodeproject.org/experiments/ENCSR270OKS/), ENCSR229JRA (https://www.encodeproject.org/experiments/ENCSR229JRA/), ENCSR910QOX (https://www.encodeproject.org/experiments/ENCSR910QOX/), ENCSR719HRO (https://www.encodeproject.org/experiments/ENCSR719HRO/), ENCSR980UEY (https://www.encodeproject.org/experiments/ENCSR980UEY/), ENCSR741QEH (https://

www.encodeproject.org/experiments/ENCSR741QEH/), ENCSR714KDG (https://www.encodeproject.org/experiments/ENCSR714KDG/), ENCSR693CSQ (https://www.encodeproject.org/experiments/ENCSR693CSQ/), ENCSR039ICU (https://www.encodeproject.org/experiments/ENCSR039ICU/), ENCSR085HNI (https://www.encodeproject.org/experiments/ENCSR085HNI/), ENCSR236OON (https://www.encodeproject.org/experiments/ENCSR236OON/), ENCSR680AAZ (https://www.encodeproject.org/experiments/ENCSR680AAZ/), ENCSR129KCJ (https://www.encodeproject.org/experiments/ENCSR129KCJ/), ENCSR448DCX (https://www.encodeproject.org/experiments/ENCSR448DCX/), ENCSR571BML (https://www.encodeproject.org/experiments/ENCSR571BML/), ENCSR448VSW (https://www.encodeproject.org/experiments/ENCSR448VSW/), ENCSR718CDN (https://www.encodeproject.org/experiments/ENCSR718CDN/), ENCSR783BUO (https://www.encodeproject.org/experiments/ENCSR783BUO/), ENCSR482VRI (https://www.encodeproject.org/experiments/ENCSR482VRI/), ENCSR817TLH (https://www.encodeproject.org/experiments/ENCSR817TLH/), ENCSR439SPU (https://www.encodeproject.org/experiments/ENCSR439SPU/), ENCSR999ZCI (https://www.encodeproject.org/experiments/ENCSR999ZCI/), and ENCSR396GIH (https://www.encodeproject.org/experiments/ENCSR396GIH/).
The remaining data are available within the Article, Supplementary Information or Source Data file.

## Human research participants

Policy information about studies involving human research participants and Sex and Gender in Research.

| | |
|---|---|
| Reporting on sex and gender | Sex was assessed by the clinics and reported in the study. Gender was not recorded in the clinic, due to medical standard procedure and therefore could not be considered. All available patients were included. However due to the small cohort size of two patients a sex- or gender-specific analysis was not feasible. |
| Population characteristics | For PBMCs of healthy blood donors no demographics were available. The AML patients were treated with Decitabine as standard of care in the clinic in Tübingen, or within the DECIDER trial in Freiburg. The median age at sample collection was 78.5 years old (range 69-84). 60% and 40% of the patients were of male and female sex, respectively. |
| Recruitment | Healthy blood donors were obtained from the blood bank after informed consent, selected for the HLA alleles of the tested t-neopeptides. AML samples were obtained from patients that were treated with Decitabine as standard of care, or within the DECIDER trial, and blood was obtained after informed consent, no patient selection was performed as all available donors were used. |
| Ethics oversight | The study was performed according to the guidelines of the ethics committee at the medical faculty of the Eberhard-Karls-University and at the University Hospital Tübingen (713/2018B02, 406/2019BO2). |

Note that full information on the approval of the study protocol must also be provided in the manuscript.

# Field-specific reporting

Please select the one below that is the best fit for your research. If you are not sure, read the appropriate sections before making your selection.

☒ Life sciences ☐ Behavioural & social sciences ☐ Ecological, evolutionary & environmental sciences

For a reference copy of the document with all sections, see nature.com/documents/nr-reporting-summary-flat.pdf

# Life sciences study design

All studies must disclose on these points even when the disclosure is negative.

| | |
|---|---|
| Sample size | Sample sizes were determined based on prior knowledge of good sample sizes to ensure adequate data for reliable assessments as well as feasibility sequencing data generation. For sequencing data, sample sizes can be extracted from the figure itself (hierachical clustering or principal component analysis). For the remaining analysis, sample sizes are indicated in the figure legends. |
| Data exclusions | Data was excluded based on QC results for individual assays. All RNA samples reached an RNA integrity number (RIN) > 8.5. For the ELISpot assay donors with no observable spot counts in the positive control (PHA-p) were excluded from the analysis. |
| Replication | All experiments were performed in at least three biological replicates and specific sample sizes are mentioned in the figure legends. Most experiments contain statistical analysis and significances of the results are indicated. |
| Randomization | Mass spectrometric measurements of treated and untreated biological replicates were performed in randomized order. |
| Blinding | The investigators were not blinded to group allocation due to fixed experimental groups used for comparison. |

# Reporting for specific materials, systems and methods

We require information from authors about some types of materials, experimental systems and methods used in many studies. Here, indicate whether each material, system or method listed is relevant to your study. If you are not sure if a list item applies to your research, read the appropriate section before selecting a response.

## Materials & experimental systems

| n/a | Involved in the study |
|---|---|
| ☐ | ☒ Antibodies |
| ☐ | ☒ Eukaryotic cell lines |
| ☒ | ☐ Palaeontology and archaeology |
| ☒ | ☐ Animals and other organisms |
| ☒ | ☐ Clinical data |
| ☒ | ☐ Dual use research of concern |

## Methods

| n/a | Involved in the study |
|---|---|
| ☒ | ☐ ChIP-seq |
| ☐ | ☒ Flow cytometry |
| ☒ | ☐ MRI-based neuroimaging |

# Antibodies

Antibodies used

IgG isotype control (400202, BioLegend, San Diego, USA)
PE/Cy7 anti-human CD8 (1:400 dilution, Cat# 737661, RRID: AB_1575980, Beckman Coulter, Brea, USA)
Pacific Blue anti-human TNF (1:120 dilution, Cat# 502920, RRID: AB_528965, BioLegend)
PE anti-human IFN-γ mAB (1:200 dilution, Cat# 506507, RRID: AB_315440, BioLegend).
anti-IFNγ antibody, MabTech, Cat# 3420-3-250, RRID:AB_907283
anti-IFNγ biotinylated detection antibody, MabTech, Cat# 3420-6-250, RRID:AB_907273
The pan-HLA class I-specific mAb W6/32, the pan-HLA class II-specific mAb Tü 39, and the HLA-DR-specific mAb L243 were produced in-house.

Validation

Antibodies were purchased from the above stated companies. Antibodies are well described and published elsewhere. Information can be sought from the manufactures website under the respective catalogue number. In-house produced antibodies were validated with concentration titration by flow cytometry.

# Eukaryotic cell lines

Policy information about cell lines and Sex and Gender in Research

Cell line source(s)

NCI-H1299 (CRL-5803, ATCC), NCI-H1395 (CRL-5868, ATCC), NCI-H1651 (CRL-5884, ATCC), NCI-H2122 (CRL-5985, ATCC), NCI-H1693 (CRL-5887, ATCC), NCI-H1395 (CRL-5868, ATCC), OCI-AML2 , (ACC 99, DSMZ), OCI-AML3 (ACC 582, DSMZ), HL60 (CCL-240, ATCC), MV411 (CRL-9591, ATCC), HCT116 (CCL-247, ATCC), SW480 (CCL-228, ATCC), DLD1 (CCL-221, ATCC), A172 (CRL-1620, ATCC), U87MG (HTB-14, ATCC), LN229 (CRL-2611, ATCC), and T98G (CRL-1690, ATCC)

Authentication

All cell lines were authenticated using the Multiplex cell line authentication test by Multiplexion (Heidelberg, Germany).

Mycoplasma contamination

All cell lines were controlled for contamination using the Multiplex cell contamination test by Multiplexion (Heidelberg, Germany).

Commonly misidentified lines
(See ICLAC register)

No commonly misidentified cell lines were used.

# Flow Cytometry

## Plots

Confirm that:

☒ The axis labels state the marker and fluorochrome used (e.g. CD4-FITC).

☒ The axis scales are clearly visible. Include numbers along axes only for bottom left plot of group (a 'group' is an analysis of identical markers).

☒ All plots are contour plots with outliers or pseudocolor plots.

☒ A numerical value for number of cells or percentage (with statistics) is provided.

## Methodology

Sample preparation

Sample preparation of the individual flow cytometry experiments are described in detail in the methods part of the manuscript.

Instrument

FACS Canto II cytometer (BD Biosciences)

Software

FlowJo 10.0.8 (BD)

Cell population abundance

CD8+ cells were sorted prior to in vitro stimulation.

Gating strategy

All gating strategies are provided in the manuscript supplementary information file.

☒ Tick this box to confirm that a figure exemplifying the gating strategy is provided in the Supplementary Information.
